# Aspirin use is associated with reduced risk for recurrence of pyogenic liver abscess: a propensity score analysis

**DOI:** 10.1038/s41598-019-48017-3

**Published:** 2019-08-08

**Authors:** Jia-Sin Liu, Chen-Hsiang Lee, Seng-Kee Chuah, Wei-Chen Tai, Chia-Chi Chang, Fang-Ju Chen

**Affiliations:** 1 Department of Public Health, College of Health Science, Kaohsiung Medical University, Kaohsiung, Taiwan; 2 Division of Infectious Diseases, Kaohsiung Chang Gung Memorial Hospital, Kaohsiung, Taiwan; 3 Chang Gung University College of Medicine, Kaohsiung, Taiwan; 4 Division of Gastroenterology, Kaohsiung Chang Gung Memorial Hospital, Kaohsiung, Taiwan

**Keywords:** Risk factors, Bacterial infection

## Abstract

Aspirin possesses anti-bacterial activity that may prevent recurrence of *Klebsiella pneumoniae* pyogenic liver abscess (KP-PLA). In e*x-vivo* study, aspirin was administered before bactericidal assay against serotype K1 *K. pneumoniae*. We identified 5,912 patients with PLA who had no known pre-existing hepatobiliary diseases or malignancy in Taiwan from 1999 to 2013 from nationwide cohort study. Multivariate Cox proportional hazards regression models was used to estimate the hazard ratios [HR] for the association between aspirin use and recurrent PLA. The PLA recurrence rate in patients taking aspirin daily for 30 or more days, from 90 days before to 90 days after the first PLA episode (aspirin users), and aspirin non-users was 42.5 and 74.6 per 1,000 person-years of follow-up, respectively. The population-based study showed a HR for PLA recurrence in aspirin users of 0.50 (95% confidence interval, 0.35–0.69), relative to that in non-users, after adjustments for confounders. An *ex-vivo* study indicated that aspirin was able to significantly enhance bacterial killing by leukocytes, whether collected from diabetic patients with KP-PLA recurrence or from healthy volunteers. Our results suggest that aspirin is associated with reduced risk for PLA recurrence among Taiwanese with PLA who had no preexisting hepatobiliary diseases or malignancy.

## Introduction

Pyogenic liver abscess (PLA) is a life-threatening infectious disease and most PLA cases used to be poly-microbial infections, with *Escherichia coli* the most common causative pathogen^1^. Since the 1980s, a highly virulent strain of *Klebsiella pneumoniae* (KP) has surpassed *E. coli* and emerged as the predominant etiology of PLA in Asia^2^, the USA^3^, Europe^4^, and globally^5^. Unlike PLAs caused by *E. coli* or other bacteria that are mostly associated with biliary tract disorders^6^, PLA caused by KP (KP-PLA) is often cryptogenic and most patients with KP-PLA have diabetes mellitus^6,7^. In addition, capsule serotypes K1 and K2 were the most common strains involved in KP-PLA^8^. The exact cause for the increasing incidence of KP-PLA is still not clear but increased intestinal colonization by these KP strains was postulated^9^. A recent study from Taiwan, where KP has been the leading etiology of cryptogenic PLA, revealed a fecal KP carriage rate of 75% in healthy adults and serotype K1/K2 isolates accounted for 23% of the typeable strains^10^, suggesting that colonization by KP strains may precede invasion of the intestinal mucosa and portal circulation, followed by the development of PLA. While the short-term morbidity and mortality of PLA are currently better understood, long-term surveillance is needed to determine the rate of PLA recurrence and factors that affect recurrence.

Aspirin has been used to prevent cardiovascular disease, and, by inhibiting cyclooxygenase-mediated arachidonic acid metabolism, can reduce the synthesis of prostaglandins, which may facilitate bactericidal activity by leukocytes^11^. In safe therapeutic concentrations, sodium salicylate (SAL), the major metabolite of aspirin, can reduce capsular polysaccharide (CPS) production by up to 70%^12^. We previously reported that SAL enhanced susceptibility of KP serotype K1 to leukocyte phagocytosis by reducing CPS production^13^; moreover, we found that patients with community-acquired KP bacteremia who had recent use of aspirin were at a lower risk of acquiring KP-associated invasive syndromes in multivariate analysis^13^, suggesting a potential role for aspirin uses in preventing recurrent KP-PLA. To date, no large-scale population-based study has investigated the relationship between KP-PLA recurrence and aspirin use. Using *ex-vivo* study model and nationwide population-based database in Taiwan, our primary aim was to determine whether aspirin use in patients with KP-PLA was associated with reduced risk for recurrence, relative to population-based controls without aspirin use.

## Results

### Patient characteristics

**Figure 1:**
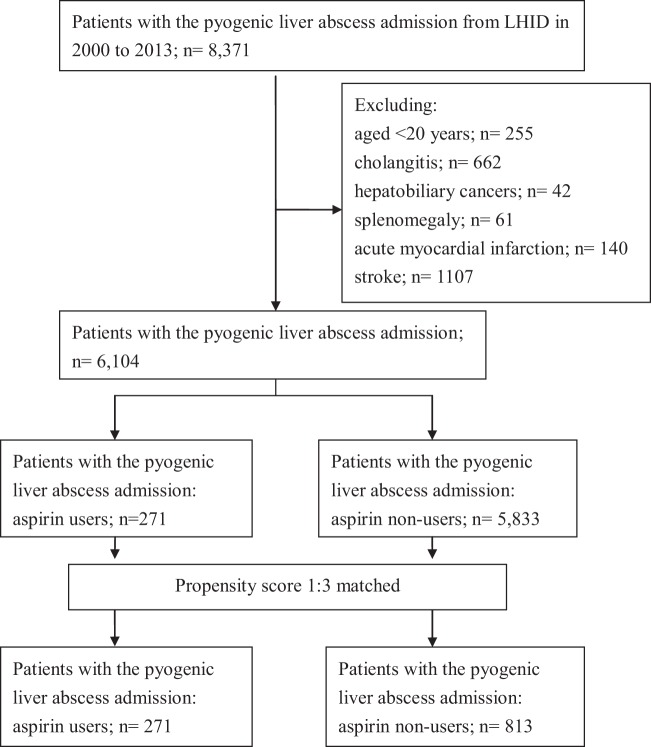
Flow chart of study cohort. Note: LHID, Longitudinal Health Insurance Database.

The medical records of 6,104 eligible patients with a first episode of PLA between 2000 and 2013 were reviewed; of these, 271 were in the aspirin-user cohort and 5,833 in the non-user cohort (Fig. 1). The demographic characteristics of both groups are summarized in Table 1. The aspirin-user group tended to be older than the non-user group. There were significant differences in sex and use of anti-diabetic medications (metformin, other OADs, and insulin), amoxicillin or ampicillin, and statins between the 2 groups. Except for cancer, with a higher rate among aspirin non-users, the aspirin-user group had higher rates of diabetic mellitus and hypertension. After 1:3 propensity-score matching, 271 aspirin users and 813 non-users were similar in terms of age, sex, baseline comorbidities, and medication use (Fig. 1).

**Table 1:** Study cohort characteristics in patients with pyogenic liver abscess.

	Before propensity-score matched				After propensity-score matched		
	Aspirin user	Aspirin non-user	*p* value	Standardized difference	Aspirin non-user	*p* value	Standardized difference
Case number	271	5,833			813		
Age group (years-old)							
20–44	6 (2.2)	1,046 (17.9)	<0.01	0.541	23 (2.8)	0.59	0.008
45–64	62 (22.9)	2,425 (41.6)	<0.01	0.408	166 (20.4)	0.39	0.045
65–74	81 (29.9)	1,113 (19.1)	<0.01	0.253	229 (28.2)	0.59	0.096
≥75	122 (45.0)	1,249 (21.4)	<0.01	0.517	395 (48.6)	0.31	0.121
Mean (sd)	71.3 (11.8)	60.1 (16.0)	<0.01	0.799	72.2 (12.4)	0.29	0.113
Gender			0.01			0.44	
Male	155 (57.2)	3,778 (64.8)		0.156	443 (54.5)		0.062
Female	116 (42.8)	2,055 (35.2)		0.156	370 (45.5)		0.062
Co-morbidity							
Diabetic mellitus	156 (57.6)	1,619 (27.8)	<0.01	0.631	427 (52.5)	0.15	0.059
Hypertension	218 (80.4)	2,030 (34.8)	<0.01	1.040	684 (84.1)	0.16	0.054
Cancer	41 (15.1)	1,449 (24.8)	<0.01	0.244	119 (14.6)	0.84	0.044
Medication use							
A moxicillin or ampicillin	26 (9.6)	348 (6.0)	0.02	0.136	72 (8.9)	0.71	0.043
Metformin	84 (31.0)	616 (10.6)	<0.01	0.520	221 (27.2)	0.23	0.056
Other oral anti-diabetic drugs	105 (38.7)	857 (14.7)	<0.01	0.564	291 (35.8)	0.38	0.069
Insulin	41 (15.1)	477 (8.2)	<0.01	0.218	94 (11.6)	0.12	0.042
Statins	53 (19.6)	236 (4.0)	<0.01	0.495	117 (14.4)	0.04	0.097
Follow-up time, year Mean (sd)	3.4 (3.5)	3.9 (4.1)	0.04	0.135	3.6 (3.7)	0.35	0.041
Propensity score, Mean (sd)	0.1237 (0.0985)	0.0407 (0.0578)	<0.01	1.021	0.1190 (0.0901)	0.46	0.046

Note: Sd, standard deviation.

### Effects of aspirin in patients with PLA recurrence

Follow-up summation was performed for 23,780 person-years of follow-up [PYFU] during the study period. The mean follow-up time was 3.4 years in aspirin users and 3.9 years in non-users. A total of 1,449 (23.7%) patients had PLA recurrence after a first episode. The PLA recurrence rate was 42.5 per 1,000 PYFU in the aspirin-user group and 74.6 per 1,000 PYFU in the non-user group. Compared with patients in the non-user group, those in the aspirin-user group had a significantly lower risk of PLA recurrence after adjustments for age, gender, diabetic, hypertension, cancer, amoxicillin or ampicillin, metformin, other OADs, insulin, statins, aspirin used (adjusted HR: 0.62; 95% CI: 0.44–0.87, *p* < 0.01); however, the differences in all-cause mortality (adjusted HR: 0.92; 95% CI: 0.75–1.13, *p* = 0.45) and risk of cancer (adjusted HR: 0.76; 95% CI: 0.54–1.08, *p* = 0.12; Table 2) were not statistically significant between the two groups.

**Table 2:** Incidence of recurrent pyogenic liver abscess events and relative incidence in patients with pyogenic liver abscess.

	Aspirin user		Aspirin non-user		Crude HR (95% confidence interval)	*p* value	Adjusted HR (95% confidence interval)	*p* value
	Incidence case	Incidence rate	Incidence case	Incidence rate				
Recurrence of pyogenic liver abscess	35	42.5	1,414	74.6	0.50 (0.35–0.69)	<0.01	0.62 (0.44–0.87)	<0.01
All-cause mortality	99	107.4	2,554	111.7	0.85 (0.70–1.04)	0.13	0.92 (0.75–1.13)	0.45
Acute myocardial infraction	6	6.8	80	3.5	1.96 (0.85–4.50)	0.11	1.45 (0.61–3.46)	0.40
Cancer	34	39.6	1,083	52.1	0.67 (0.48–0.94)	0.02	0.76 (0.54–1.08)	0.12

Incidence rate, 1000 person-years.

Note: HR, hazard ratios.

Adjusted HR: adjusted age, gender, diabetic, hypertension, cancer, amoxicillin or ampicillin, metformin, other oral anti-diabetic drugs, insulin, statins, aspirin used.

**Figure 2:**
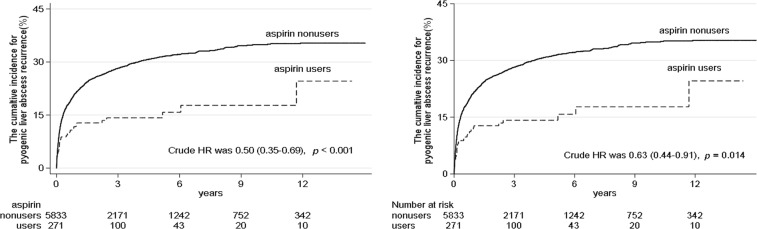
(**A**) Cumulative proportion of patients developing recurrent pyogenic liver abscess events during follow-up according to aspirin uses and non-uses, before propensity-score matching. Kaplan-Meier analysis for cumulative incidence of pyogenic liver abscess recurrence showed significantly lower risk in the aspirin users group than in the non-users group before propensity-score matching (*p* < 0.001). (**B**) Cumulative proportion of patients developing recurrent pyogenic liver abscess events during follow-up according to aspirin users and non-users, after propensity-score matching. Kaplan-Meier analysis for cumulative incidence of pyogenic liver abscess recurrence showed significantly lower risk in the aspirin users group than in the non-users group after propensity-score matching (*p* = 0.014).

For development of the propensity score, a logistic regression model was applied (Table S1). In the propensity-score matched and competing adjusted-risk model, aspirin users still had a lower risk of PLA recurrence, with an adjusted HR of 0.65 (95% CI: 0.45–0.95; *p* = 0.02; Table 3). Meanwhile, comorbidity with diabetes or hypertension and use of metformin or insulin were also risk factors for PLA recurrence (Table 3). The Kaplan-Meier analysis for cumulative incidence of PLA recurrence showed a significantly lower risk of recurrent PLA in the aspirin-user group compared with that in the non-user group both before propensity-score matching (*p* < 0.001; Fig. 2A) and after propensity-score matching (*p* = 0.014; Fig. 2B).

**Table 3:** Factors associated with recurrent pyogenic liver abscess events from multivariate analysis.

	Crude model		Adjusted model		Adjusted model after PS matched		Competing risk Adjusted model after PS matched	
	HR (95%CI)	*p* value	HR (95%CI)	*p* value	HR (95%CI)	*p* value	HR (95%CI)	*p* value
Aspirin	0.50 (0.35–0.69)	<0.01	0.62 (0.44–0.87)	<0.01	0.65 (0.45–0.95)	0.02	0.62 (0.44–0.88)	<0.01
Age group (years-old)								
20–44	0.91 (0.79–1.04)	0.17	0.87 (0.69–1.08)	0.20	0.31 (0.09–1.06)	0.06	0.86 (0.69–1.07)	0.18
45–64	0.92 (0.80–1.06)	0.27	1.07 (0.88–1.32)	0.49	1.48 (0.82–2.66)	0.19	1.07 (0.87–1.31)	0.51
65–74								
75+	0.65 (0.56–0.76)	<0.01	0.83 (0.61–1.13)	0.23	1.38 (0.56–3.35)	0.48	0.79 (0.58–1.08)	0.14
Male vs. Female	1.38 (1.23–1.54)	<0.01	1.32 (1.18–1.48)	<0.01	1.46 (1.07–1.99)	0.02	1.24 (1.11–1.39)	<0.01
Co-morbidity								
Diabetes	1.08 (0.97–1.21)	0.16	1.35 (1.16–1.57)	<0.01	1.53 (1.01–2.30)	0.04	1.32 (1.14–1.54)	<0.01
Hypertension	0.86 (0.77–0.96)	<0.01	0.98 (0.87–1.11)	0.80	0.83 (0.58–1.18)	0.30	0.99 (0.87–1.13)	0.91
Cancer	1.77 (1.57–1.99)	<0.01	1.78 (1.57–2.01)	<0.01	1.79 (1.22–2.64)	<0.01	1.10 (0.98–1.25)	0.12
Prescribed								
Amoxicillin or ampicillin	0.54 (0.42–0.71)	<0.01	0.56 (0.43–0.73)	<0.01	0.78 (0.45–1.35)	0.37	0.61 (0.47–0.80)	<0.01
Metformin	0.76 (0.64–0.90)	<0.01	0.65 (0.53–0.80)	<0.01	0.59 (0.40–0.88)	<0.01	0.67 (0.55–0.83)	<0.01
Other oral anti- diabetic drugs	0.99 (0.87–1.14)	0.92	1.15 (0.95–1.39)	0.15	1.09 (0.74–1.62)	0.65	1.23 (1.02–1.48)	0.03
Insulin	1.00 (0.83–1.20)	0.99	0.92 (0.76–1.13)	0.44	0.98 (0.63–1.50)	0.91	0.94 (0.77–1.15)	0.54
Statins	0.54 (0.40–0.74)	<0.01	0.63 (0.46–0.86)	<0.01	0.56 (0.35–0.90)	0.02	0.67 (0.49–0.92)	0.01

Note: HR, hazard ratios.

The aspirin users in subgroup analysis had lower competing adjusted risk for PLA recurrence after a first episode of PLA; this was especially significant for females, those with diabetes or a non-cancer comorbidity, amoxicillin and ampicillin non-users, other anti-diabetic drug non-users, and insulin non-users (Fig. S1). Cause-specific hazard analysis also yielded similar results (data not shown). Aspirin use in the 6–12 months and 3–6 months prior to a first episode of PLA did not show a lower risk for PLA recurrence. The HR for PLA recurrence among patients with aspirin use at the time of the first episode of PLA was 0.62 (95% CI: 0.41–0.93; *p* = 0.02), and the HR for PLA recurrence among patients with aspirin use after the first episode was 0.98 (95% CI: 0.97–0.98, *p* < 0.01), with both groups having a lower risk of PLA recurrence (Table 4).

**Table 4:** Estimated hazard ratio for pyogenic liver abscess recurrence with use of aspirin for various durations.

Aspirin used time	aspirin users	aspirin non-users	Adjusted HR	*p* value
Prior 6–12 months before first episode of PLA	558	5,546	1.28 (0.98–1.68)	0.07
Prior 3–6 months before first episode of PLA	452	5,652	0.89 (0.64–1.25)	0.52
During first episode of PLA	271	5,833	0.62 (0.41–0.93)	0.02
After first episode of PLA	1,251	4,853	0.98 (0.97–0.98)	<0.01

Note: HR, hazard ratios; PLA, pyogenic liver abscess.

Adjusted HR: adjusted age, gender, diabetic, hypertension, cancer, amoxicillin or ampicillin, metformin, other oral anti-diabetic drugs, insulin, statins, aspirin used and aspirin used after pyogenic liver abscess first even as a time-dependent covariate.

**Figure 3:**
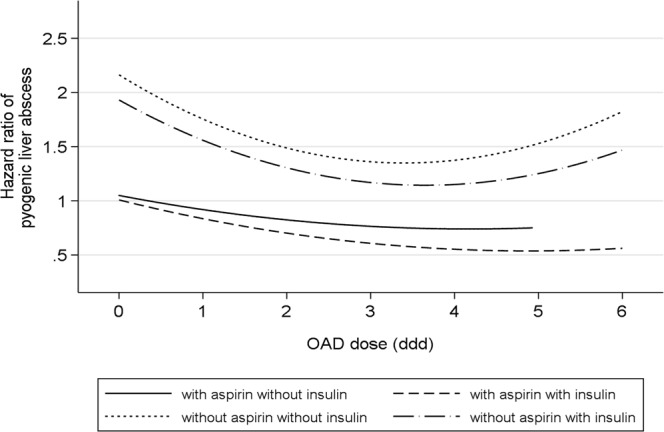
Dose-response association between oral anti-diabetic drug use (daily defined dose) and hazard ratio for recurrent pyogenic liver abscess in diabetic patients. Aspirin use was associated with lower risk for recurrent pyogenic liver abscess, especially in diabetic patients who did not receive an oral anti-diabetic drug (OAD) or those who received a high OAD dose for sugar control. Aspirin use did not show a significant difference in risk for recurrent pyogenic liver abscess in a comparison between diabetic patients with and without insulin therapy. Note: ddd, daily defined dose.

The restricted cubic spline Cox model indicated that aspirin use was associated with a lower recurrent risk for PLA, especially in diabetic patients who did not receive an OAD or those who received a high-dose OAD for glucose control (Fig. 3). Aspirin use did not show a significant difference in risk for PLA recurrence in diabetic patients with and without insulin therapy.

### Effects of aspirin on *ex vivo* leukocyte bactericidal activity

Of 5 diabetic patients admitted to KCGMH between 2013 and 2016 with a diagnosis of recurrent KP-PLA, 3 were males and 2 females, with a mean age of 59.8 years (range: 54–68 years). These 5 patients did not take aspirin before or after a first episode of KP-PLA. The average time between first occurrence and KP-PLA recurrence was 1.7 years (range: 0.8–2.4 years). Poor glycemic control with hemoglobin A1c above 9.0% (average: 10.7%; range: 9.4–12.5%) was observed in these 5 patients. The demographic information and data gathered from these patients are shown in Table S2.

**Figure 4:**
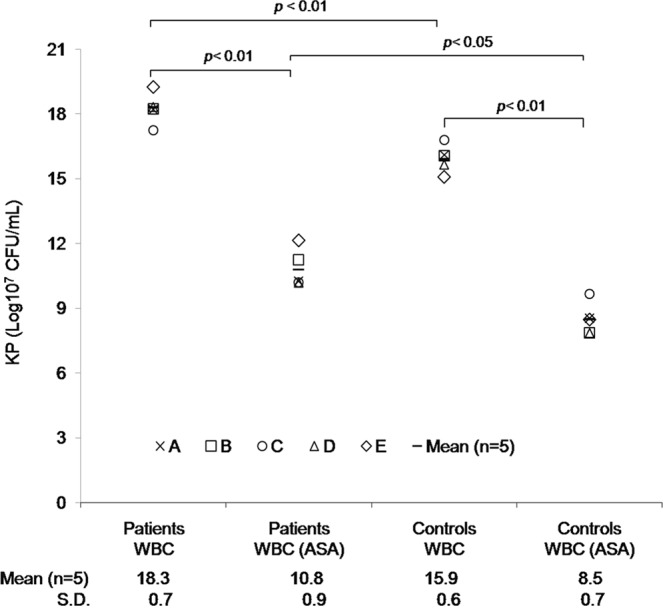
*Ex-vivo* human leukocyte bactericidal activity assay. Peripheral WBCs from 5 diabetic patients with recurrent pyogenic liver abscess caused by KP and 5 healthy volunteers (controls) were collected before and 1 h after oral administration of 100 mg of ASA, and were incubated with KP that had previously been opsonized with normal human serum. ASA treatment significantly enhanced leukocyte killing of KP (*p* < 0.01), regardless of whether leukocytes were collected from patients or healthy volunteers. Note: ASA, aspirin; KP, *Klebsiella pneumoniae;* WBC, white blood cells.

The difference between the rate of KP killing by leukocytes from these 5 diabetic patients and 5 healthy volunteers before oral administration of 100 mg of aspirin was significant (*p* < 0.01; Fig. 4). KP was significantly more susceptible to killing after incubation with leukocytes from healthy volunteers. The *ex vivo* bactericidal activity of leukocytes collected 1 h following oral administration of 100 mg of aspirin from 5 diabetic patients with KP-PLA recurrence and 5 healthy male volunteers was significantly greater than that of leukocytes collected from the same patients or from volunteers before aspirin treatment (*p* < 0.01; Fig. 4). The results indicated that aspirin was able to significantly enhance bacterial killing by leukocytes, whether collected from diabetic patients with KP-PLA recurrence or from healthy volunteers.

## Discussion

We provided evidence that exposure to aspirin for more than 30 days, from 90 days before to 90 days after the first episode, was associated with decreased risk for recurrence of PLA in a nationwide population-based study in Taiwan. The duration relationship for aspirin use (both at the time of the first episode and at the time after the first episode of PLA; Table 4) was evident in the current study. Furthermore, the effect of aspirin use on a lower risk for PLA recurrence was found especially in diabetic patients who did not receive an OAD or in those on a higher OAD dose for glucose control (Fig. 4). The risk of recurrence may vary with glycemic status in diabetic patients; however, no laboratory tests that could serve as a proxy for glycemic status (HbA1c) were available in this dataset. Our findings imply that, with aspirin use, PLA may be preventable, especially in patients with a new diagnosis of diabetes and in diabetic patients with poor glucose control. We further demonstrated that aspirin enhanced leukocyte bactericidal activity against KP in diabetic patients with KP-PLA recurrence and in healthy volunteers in an *ex-vivo* study. To our knowledge, this is the first longitudinal study that specifically examined the role of aspirin in the prevention of PLA recurrence, using both population-based and *ex-vivo* findings.

Recurrent KP-PLA has seldom been reported^14,15^. Recently, a tertiary teaching hospital in China reported that 4 (4.1%) of 98 cases of KP-PLA had a prior history of PLA; therefore, these 4 cases were thought to represent recurrences, with onset between 7 and 96 months after the first episode^16^. In another study conducted in Taiwan that included 601 patients with PLA with a mean follow-up of 6 years, the cumulative recurrence rate of PLA was lower in both the patients with cryptogenic PLA (2.0%) and diabetic patients with PLA (4.4%) than those pre-existing hepatobiliary diseases with PLA (23.8%)^1^; moreover, 80% of diabetic patients initially infected with KP in this study were again infected with KP when the PLA recurred, which suggests that the recurrence rate of PLA in diabetic patients was low and most PLA recurrence was due to KP^1^. However, these single-center retrospective studies were limited by selection and recall bias^1,16^, and our large-scale population-based study might overcome these limitations. Furthermore, our cohort excluded patients with cholangitis, hepatobiliary cancer, or splenomegaly to reduce the complexity of analysis. Meanwhile, we also excluded patients with prior myocardial infarction or stroke to ensure comparability between aspirin users and non-users. The results showed that aspirin therapy decreased the risk of PLA recurrence. This finding was also confirmed in multivariate analysis after adjusting for a number of potential confounders, which was further reinforced by the duration relationship for aspirin use.

Adding to the long list of benefits, researchers have found that aspirin can reduce virulent bacteria associated with serious infections. One study found that SAL, a byproduct of aspirin, disrupted bacterial ability to adhere to host tissue, reducing propagation and spread of infection. Animals treated with aspirin have fewer bacteria at sites of infection, and develop smaller abscesses^17^. Additionally, our previous study has demonstrated that SAL could affect CPS production in KP, therefore attenuating the pathogenicity of KP strains. Aspirin also promotes human leukocyte phagocytosis and bactericidal activity against KP^13^. The majority of KP-PLA cases are preceded by colonization of the gastrointestinal tract, as suggested in epidemiological studies^9^. We found that patients who had used aspirin during the month prior to the diagnosis of community-acquired KP bacteremia appeared to have a lower risk for invasive syndromes including PLA^13^. As a result, aspirin may not be curative, but it could reduce the ability of bacteria to cause infection. We speculate that, with time, the immune system can adapt to compensate for or overcome the deficits, thereby decreasing the risk of PLA associated with use of aspirin. More studies are needed to confirm this hypothesis. The *ex-vivo* study still suggests that aspirin renders the host susceptible to KP. However, aspirin may simply arrest invasion by KP through the loss of resistance to colonization by KP. The consequences of an altered intestinal microbiota due to aspirin therapy are not fully understood. We did not explore how aspirin administration disturbs host-microbiota homeostasis that leads to KP-PLA. This also presents an opportunity for intervention, if established intestinal colonization can be eliminated. Use of aspirin to enhance innate immune defenses and inhibit pathogen colonization of the intestinal lumen and translocation across mucosal barriers may provide new strategies for management of this emerging infectious disease.

This study had some limitations. First, this was an observational study and could not prove a causal relationship. However, the *ex-vivo* leukocyte bactericidal experiment showed that aspirin was able to significantly enhance bacterial killing by leukocytes, subsequently resulting in lower risk for PLA recurrence. Second, smoking, drinking, exercise habits, diet, and other behaviors were not included in our model, but these may influence the relationship between aspirin and PLA recurrence. To minimize bias, we used propensity-score matching and subgroup analyses, which showed a strong relationship with health-seeking behaviors, but still indicated that aspirin was associated with reduced risk for PLA recurrence. Third, the disease and drug records collected from the databases may have been incorrect. We considered PLA as a critical event. Although, and used ICD-9-CM code 041.3 to identify sources of infection other than the lung. This code may have been omitted in the dataset. Our PLA cohort excluded patients with a history of hepatobiliary malignancy, cholangitis, common bile duct stones, and portal hypertension with splenomegaly to limit PLA to cryptogenic cases^18^.

In conclusion, our *ex-vivo* study suggested that aspirin enhanced the bactericidal activity of leukocytes against KP. The translation of these insights into the clinical arena demonstrated that exposure to aspirin was associated with reduced risk for PLA recurrence; moreover, a duration relationship was evident in the population-based study. Future studies are warranted to clarify the mechanism by which aspirin alters the pathogenesis of PLA by KP and how aspirin influences the risk of KP-PLA recurrence.

## Methods

### Data sources

The present study used data from the Taiwan National Health Insurance Research Database (NHIRD). The NHIRD, initiated in 1995, has been managed and released to the public by the National Health Research Institute (NHRI) of Taiwan, and presently covers more than 99% (approximately 23 million) of the residents in Taiwan. For use in research and health policy development, the Taiwan NHRI created a longitudinal health insurance database (LHID) that included 1 million randomly sampled residents in 2005. There were no significant differences in characteristics, including age and sex, between enrolled residents in the LHID and those in the NHIRD^19,20^. Any information in the LHID that could reveal the identities of individual patients was de-identified. All procedures including experimental protocol in this study followed the directives of the Declaration of Helsinki and informed consent were approved by the Institutional Review Board of Kaohsiung Chang Gung Memorial Hospital (KCGMH; 201601500B0).

We identified patients hospitalized with the diagnosis of PLA (ICD-9 code 572.0) from the LHID between January 1, 2000 and December 31, 2013. Aspirin use (Anatomical Therapeutic Chemical code B01AC06) was defined as dosing of aspirin for more than 30 days, from 90 days before to 90 days after the first PLA episode during the study period. To confirm duration relationship, we also analyzed aspirin use for 6–12 months and 3–6 months prior to the first PLA episode, as well as use after the PLA episode.

### Baseline characteristics

Baseline characteristics recorded within 3 years before the first episode of PLA included age, sex, comorbidities, and medication usage. Comorbidities of diabetes mellitus (ICD-9 codes 250, 357.2, 366.41, 362.0A181), hypertension (ICD-9 codes 401, 402, 403, 404, 405, A260, A269), and cancer (ICD-9 codes 140–208) were defined as diagnoses made at 2 outpatient visits or during 1 hospitalization within 3 years before the first episode of PLA. Prescriptions for amoxicillin or ampicillin, metformin, other oral anti-diabetes drugs (OADs) (sulfonylureas, alpha-glucosidase inhibitors, thiazolidinediones, dipeptidyl peptidase-4 inhibitors), insulin, and statins were also defined as dosing for more than 30 days, from 90 days before to 90 days after the first episode; these were treated as covariates in this study.

Those who did not receive aspirin during the study period were considered controls and were assigned an index date corresponding to the same year of the case. Patients younger than 20 years and those with prior stroke or acute myocardial infarction were excluded. The etiology of cryptogenic PLA was nearly always KP in Taiwan^18^. Therefore, we further excluded patients with a history of hepatobiliary malignancy, cholangitis, common bile duct stones, or portal hypertension with splenomegaly in order to limit our cohort to PLA caused by KP. Based on intention-to-treat principle, analyses were conducted according to initial assignment of aspirin modality, regardless of any subsequent changes.

### Outcome variables: PLA recurrence, complications, and mortality

The observation period started 30 days after discharge for the first episode of PLA and ended when the patient died or developed recurrence, or December 31, 2013, which ever occurred earlier. The incidence of cancer or myocardial infarction was also followed up until death or December 31, 2013. Mortality records were retrieved from patients who died in the hospital or withdrawn from the NHIRD.

### Study design and participants for *ex-vivo* human leukocyte bactericidal activity assay

Diabetic patients admitted to KCGMH between January 1, 2013 and December 31, 2016 with community-acquired mono-microbial bacteremia caused by KP were included to determine the patients with KP-PLA recurrence^21^. PLA recurrence was defined as having a typical clinical presentation, with new imaging findings of abscess recurrence after the first episode of abscess had fully resolved. All isolated KP strains were stored at −80 °C before use. Peripheral blood samples were collected from 5 healthy male volunteers aged 25–40 years and from participants with recurrent KP-PLA for an *ex-vivo* bactericidal assay as described below. All participants provided written informed consent.

### Determination of *K. pneumoniae* serotype and *rmpA* genes

Genotyping of 7 clinically significant capsular types (K_1_, K_2_, K_5_, K_20_, K_54_, K_57_, and K_N1_) for all KP isolates was performed with a polymerase chain reaction (PCR) assay using primers designed for the *cps* variable region^22^. PCR was also used to determine the presence of *rmpA*. Previously published primers for *rmpA* were used, and the expected PCR products of *rmpA* were 535 bp in length^23^.

### *Ex-vivo* human leukocyte bactericidal activity assay

Bactericidal activity was measured using a standard assay method^24^. The KP isolate from each enrolled patient during the first episode of KP-PLA (study for each patient, respectively) and serotypes K1 KP, KP-M1 (study for volunteers) were cultured on Tryptic Soy Agar medium overnight at 37 °C, and then opsonized with addition of 10% pooled serum collected from 5 healthy male volunteers who did not take any aspirin prior to donation. The suspension was mixed at a 10:1 ratio of infected to whole blood leukocytes, which had been collected from diabetic patients with recurrent KP PLA and 5 volunteers, either before or 1 h after a 100-mg oral dose of aspirin. Samples were collected 1 h later and diluted in H_2_O (pH 11.0, adjusted with NaOH) to lyse the leukocytes and disperse the bacteria for the power-plate colony assay^25^. All tests were performed in triplicate to ensure reproducibility.

### Statistical analysis

Baseline characteristics were compared using either a 2-sided t-test or χ^2^ test as suitable. In multivariate Cox proportional hazards regression models, the effects of aspirin were adjusted for age, sex, comorbidities, and prescriptions used. Results were expressed as hazard ratios (HRs) and compared with those aspirin non-users. All time-independent covariates were compared using estimated log-log survival curves to ensure a constant HR over time. Adjusted HRs and 95% confidence intervals (CIs) for PLA recurrence with aspirin use were based on participant characteristics (see below). After adjusting for covariates, the cumulative hazards of PLA recurrence over time were compared between aspirin users and non-users with Cox proportional hazards regression models and a cause-specific, PLA recurrence and mortality, competing adjusted-risk model^26^. Propensity-score matched logistic regression analysis for the likelihood of aspirin use was performed using the baseline covariates. Adequacy of balance for the covariates in the matched sample was assessed using the standardized mean difference between the 2 groups, with differences less than 10% reflecting good balance.

In subgroup analysis for subjects with diabetes, restricted cubic spline curves were used to examine non-linear associations of aspirin users and non-users with recurrent PLA, with adjustments for confounding variables, to further characterize the nature of the relationships between OAD doses with the risk of PLA recurrence. Four knots were chosen to produce a curve that appeared adequately smooth^27^. To control for consistent medication effect across different durations, the aspirin used after the first episode of PLA was assessed as a duration relationship covariate in the Cox model. The counts of surviving bacteria in the bactericidal activity assay were analyzed using the log rank test. The 2-tailed test was used in statistical significance testing and a *p* value < 0.05 was considered significant. All statistical analyses were carried out using STATA 15.1 (STATA Corp., College Station, TX, USA) and SAS 9.4 (SAS Institute Inc., Cary, NC, USA).

## Supplementary information

Aspirin use is associated with reduced risk for recurrence of pyogenic liver abscess: a propensity score analysis
