# Supplementary material for: Aspirin use is associated with reduced risk for recurrence of pyogenic liver abscess: a propensity score analysis
Source: Sci Rep. 2019 Aug 8;9:11511. doi: 10.1038/s41598-019-48017-3 (PMC6687709; doi:10.1038/s41598-019-48017-3)
Supplement: Supplementary file 1 — Aspirin use is associated with reduced risk for recurrence of pyogenic liver abscess: a propensity score analysis [file 41598_2019_48017_MOESM1_ESM.pdf]

Aspirin use is associated with reduced risk for recurrence of pyogenic liver abscess: a propensity score analysis

Jia-Sin Liu[1], Chen-Hsiang Lee[2,3], Seng-Kee Chuah[3,4], Wei-Chen Tai[3,4], Chia-Chi Chang[2] & Fang-Ju Chen[2]

[1]Department of Public Health, College of Health Science, Kaohsiung Medical University, Kaohsiung, Taiwan. [2]Division of Infectious Diseases, Kaohsiung Chang Gung Memorial Hospital, Kaohsiung, Taiwan. [3]Chang Gung University College of Medicine, Kaohsiung, Taiwan. [4]Division of Gastroenterology, Kaohsiung Chang Gung Memorial Hospital, Kaohsiung, Taiwan.

Correspondence and requests for materials should be addressed to C. H. L. (lee900@adm.cgmh.org.tw)

Figure S1. Competing risk adjusted model for recurrent pyogenic liver abscess: subgroup analysis.

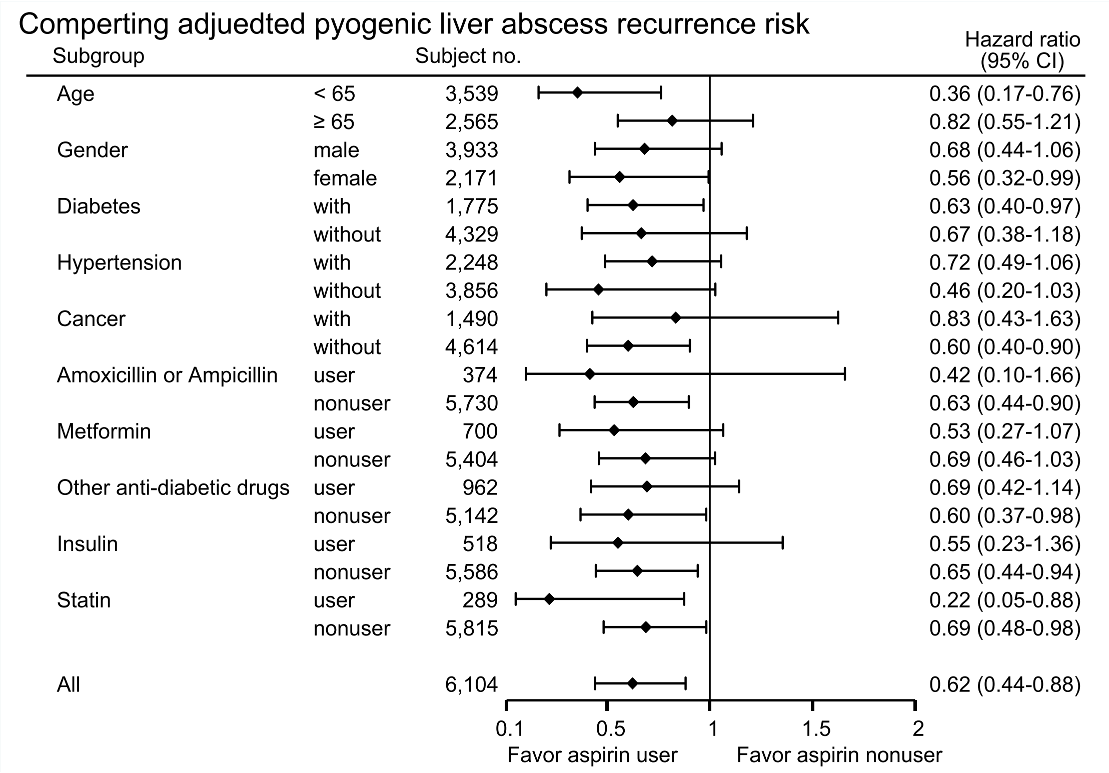

In subgroup analysis, aspirin users who were female, those with diabetes or non-cancer comorbidities, amoxicillin and ampicillin non-users, other anti-diabetic drug non-users, and insulin non-users had a significantly lower risk for pyogenic liver abscess recurrence after a first episode.

Table S1. The logistic model of propensity score for used and non-used aspirin.

Variable	Odd ratio (95% confidence interval)	*p* value
Age group (years-old)		
20-44	0.49 (0.27-0.88)	0.02
45-64	Reference	
65-74	1.63 (1.18-2.26)	<0.01
75+	2.04 (1.25-3.34)	<0.01
Male vs. Female	1.36 (1.15-1.62)	< 0.01
Co-morbidity		
Diabetes	1.27 (1.01-1.60)	0.04
Hypertension	4.09 (3.35-4.99)	< 0.01
Cancer	0.52 (0.42-0.65)	< 0.01
Prescribed		
Amoxicillin or ampicillin	1.45 (1.08-1.95)	0.01
Metformin	1.72 (1.36-2.17)	< 0.01
Other anti- diabetic drugs	1.60 (1.27-2.02)	< 0.01
Insulin	1.14 (0.88-1.47)	0.33
Statins	2.92 (2.29-3.73)	< 0.01

Table S2. Demographic data and clinical presentations of diabetic patients with recurrent *Klebsiella pneumoniae* liver abscess for *ex vivo* leukocyte bactericidal activity.

	**Case 1**	**Case 2**	**Case 3**	**Case 4**	**Case 5**
**Sex/Age (y)**	M/64	M/55	F/68	M/54	F/58
**Underlying diseases**	Diabetes, Hypertension	Diabetes, Chronic renal failure, Gout	Diabetes	Diabetes, Hypertension	Diabetes, Hypertension
**HbA1c (%)**	12.5	9.8	10.2	9.4	11.8
**Treatment of diabetes**	Insulin	Metformin, Dipeptidyl peptidase-4	Insulin, Metformin	Thiazolidinediones, Sulfonylureas, Metformin	Insulin, Sulfonylureas, Metformin

		inhibitors			
**Interval of onset (y)**	0.8	2	1.5	2.4	2
**Serotype of *K. pneumoniae* isolates (1st)**	K1	K1	K1	K1	K1
rmpA	Positive	Positive	Positive	Positive	Positive
**Serotype of *K. pneumoniae* isolates (2nd)**	K1	K1	K1	K1	K1
rmpA	Positive	Positive	Positive	Positive	Positive
**1st abscess**					
**size (cm)**	4.8	6	3	3	9
**Site (segment)**	4	3	3	4	6 & 7

**Bacteremia**	Yes	Yes	Yes	Yes	Yes
**Drainage**	Yes	Yes	No	No	Yes
**Metastatic complications**	Lung abscess	Endophthalmitis	None	None	Renal abscess
**Recurrent abscess**					
**size (cm)**	2.5	8	7	2	5
**Site (segment)**	6	6	6 & 7	6	4
